# Discovery and characterisation of a novel toxin from *Dendroaspis angusticeps*, named Tx7335, that activates the potassium channel KcsA

**DOI:** 10.1038/srep23904

**Published:** 2016-04-05

**Authors:** Iván O. Rivera-Torres, Tony B. Jin, Martine Cadene, Brian T. Chait, Sébastien F. Poget

**Affiliations:** 1 Department of Chemistry, CUNY Graduate Center and Institute for Macromolecular Assemblies, College of Staten Island, City University of New York, Staten Island, NY 10314, USA; 2 LaGuardia Community College, City University of New York, Long Island City, NY 11101, USA; 3 The Rockefeller University, New York, NY 10065, USA.

## Abstract

Due to their central role in essential physiological processes, potassium channels are common targets for animal toxins. These toxins in turn are of great value as tools for studying channel function and as lead compounds for drug development. Here, we used a direct toxin pull-down assay with immobilised KcsA potassium channel to isolate a novel KcsA-binding toxin (called Tx7335) from eastern green mamba snake (*Dendroaspis angusticeps*) venom. Sequencing of the toxin by Edman degradation and mass spectrometry revealed a 63 amino acid residue peptide with 4 disulphide bonds that belongs to the three-finger toxin family, but with a unique modification of its disulphide-bridge scaffold. The toxin induces a dose-dependent increase in both open probabilities and mean open times on KcsA in artificial bilayers. Thus, it unexpectedly behaves as a channel activator rather than an inhibitor. A charybdotoxin-sensitive mutant of KcsA exhibits similar susceptibility to Tx7335 as wild-type, indicating that the binding site for Tx7335 is distinct from that of canonical pore-blocker toxins. Based on the extracellular location of the toxin binding site (far away from the intracellular pH gate), we propose that Tx7335 increases potassium flow through KcsA by allosterically reducing inactivation of the channel.

The potassium ion (K^+^) channel from *Streptomyces lividans* (KcsA) is a member of the family of ion-selective pores called tetrameric cation channels and the archetype for a K^+^-selective ion channel pore^1^,^2^. K^+^ channels are highly relevant to various biological processes such as cardiac and neuronal electrical signalling, and their malfunction has been linked to diseases such as cardiac^3^ and neuronal^4^,^5^,^6^ disease and cancer^7^. The study of K^+^ channels, and in particular the study of how K^+^ permeation through the membrane is regulated in these channels, therefore offers the potential to develop therapeutic applications towards a large range of important human diseases.

Ion flow through K^+^ channels is generally regulated by two processes: activation in response to a stimulus (voltage, ligand binding), and inactivation from the activated state in a stimulus-independent manner^8^,^9^. In KcsA, which is gated by pH, the activation gate is controlled by a number of ionisable residues on the cytosolic side of the channel. Upon protonation at low pH, several critical ionic interactions are lost, leading to an outward movement of the C-terminal helix (TM2) of each of the four subunits and the opening of a pathway for the potassium ions^10^,^11^,^12^,^13^. However, channels opened by lowering of the pH stop conducting potassium ion currents within 1–3 seconds due to slow (or C-type) inactivation, a conformational transition happening at the level of the selectivity filter^14^,^15^,^16^,^17^, the narrowest part of the ion permeation pathway.

In spite of this mechanistic knowledge of conformational gating in KcsA, the availability of further tools for directly studying the regulation of inactivation would be very useful. Animal peptide toxins may serve as such tools and have been used to study functional and regulatory aspects of channel behaviour, but their usefulness is somewhat reduced by the fact that most toxins affecting potassium channels act as pore blockers, thus inhibiting potassium flow^18^. Indeed, an NMR structure of KcsA bound to the antagonist scorpion toxin charybdotoxin reveals that this toxin binds to KcsA without inducing any structural changes, instead making specific contacts with the extracellular surface of the ion channel that result in pore blockage^19^. This lock-and-key mechanism of toxin block has been confirmed in a recent crystal structure of the same toxin in complex with a eukaryotic voltage-gated potassium channel^20^. Therefore, the availability of toxins acting through a different mechanism or having an activating effect would greatly enhance the tool kit of K^+^ channel mechanistic research.

One potential source for K^+^ channel toxins are the venoms of mamba snakes, which contain two main groups of neurotoxins. The three-finger toxins mainly act upon ligand-gated channels and G protein coupled receptors; and the dendrotoxins target K^+^ channels^21^. The dendrotoxins are small proteins, containing 57–60 amino acid residues cross-linked by three disulphide bridges. Although they adopt a Kunitz-type protease inhibitor fold, they show little or no anti-protease activity but block particular subtypes of voltage-dependent potassium channels of the Kv1 subfamily in neurons^22^. Studies with cloned K^+^ channels demonstrated that α-dendrotoxin from *Dendroaspis angusticeps* blocks voltage-gated Kv1.1, Kv1.2 and Kv1.6 channels in the nanomolar range, whereas toxin K from *Dendroaspis polylepis* preferentially blocks Kv1.1 channels^22^. The details of the pore blocking effect of δ-dendrotoxin on a *shaker*-type potassium channel have been functionally elucidated by mutant-cycle analysis^23^, and a structural model showing presumed binding contacts for the pore blocker δ-dendrotoxin in complex with this channel was generated^24^. However, to our knowledge no channel-activating toxins from mamba venom have been reported so far.

Here, we present the discovery, isolation and functional characterisation of a distinct channel-opener toxin obtained from eastern green mamba venom. This toxin, named Tx7335, increases channel opening events in a dose-dependent manner if added to the extracellular side of a planar bilayer system with reconstituted KcsA single channels. This observation means that the channel-opener toxin does not directly affect the intracellular pH gate, and the likely mode of action is through a shift in equilibrium towards the conductive state of the inactivation gate. The discovery of this toxin offers a unique experimental tool to study the allosteric regulation of inactivation in KcsA. It also offers the opportunity for further studying the underlying conformational and dynamic processes involved in potassium channel inactivation through the use of a direct agonist peptide.

## Results

### Toxin pull-down assay and identification of Tx7335

**Figure 1:**
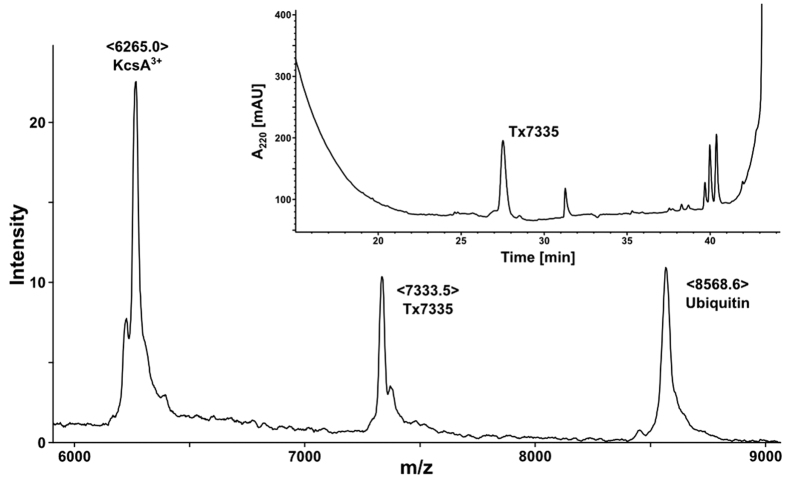
Pull-down of Tx7335 with immobilised KcsA as shown by MALDI and HPLC analysis. The main spectrum shows a MALDI mass spectrum of KcsA eluted from a metal affinity resin column after incubation with *D. angusticeps* venom. The peaks correspond to the triply-charged KcsA species, to the ubiquitin used as an internal calibrant and to the novel toxin Tx7335 that was pulled down by the channel. The insert shows an HPLC chromatogram of the purification of Tx7335 from the affinity column eluate via reverse-phase chromatography.

In order to identify toxins in *Dendroaspis angusticeps* (eastern green mamba) venom that interact with KcsA, we set up toxin pull-down experiments from crude venom with immobilised KcsA. A Co^2+^-based affinity resin was used to immobilise the polyhistidine-tagged full-length KcsA in n-decyl maltoside (DM) micelles. After incubation of KcsA resin and several washing steps, any bound toxins were released by elution of the channel with a high imidazole buffer. In initial experiments, the negative control pull-down performed with KcsA-free Co^2+^-resin showed that even after extensive washes, a significant number of false positives remained in the elution buffer. Therefore, we proceeded to pre-depleting the crude venom of most non-specific binders by eluting it over free Co^2+^-resin before using it in toxin pull-down experiments. With this pre-depletion step, false positives were no longer observed, and a single, previously unknown toxin with an observed molecular mass of 7333.5 Da was identified as a specific binder by mass spectrometry and HPLC analysis (Fig. 1). This toxin was named Tx7335 (based on the theoretical mass of the subsequently derived amino acid sequence taking into account the presence of four disulphide bonds). Tx7335 was also pulled down when we used the Q58A, T61S, R64D KcsA triple mutant (data not shown). This mutant form of KcsA, referred to as pmut3, had been generated to mimic the outer pore of eukaryotic Kv1 family members and is able to bind pore-blocker toxins like charybdotoxin and agitoxin^25^. The fact that Tx7335 binds both wild-type and pmut3 KcsA suggests that this toxin has a mechanism of action that differs from the classical pore-blocker toxins.

### Tx7335 direct purification from crude venom by HPLC chromatography

In order to obtain sufficient quantities of Tx7335 for further characterisation, we directly purified the toxin from crude *Dendroaspis angusticeps* venom via HPLC using a reverse-phase C-18 column. We collected the fraction at the Tx7335 retention time and confirmed the identity of the purified toxin by measuring its mass and observing its ability to bind to KcsA immobilised on Co^2+^-resin as above.

### Determination of amino acid sequence of Tx7335

**Figure 2:**
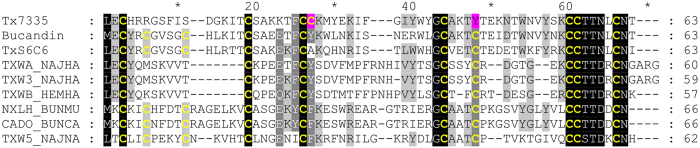
Sequence alignment of Tx7335 with other three-finger toxins. The sequence conservation at individual positions in the alignment is indicated by the darkness of the shading. Cysteine residues are printed in bold yellow. The residues differing from the canonical three-finger toxin cysteine scaffold are highlighted in magenta in Tx7335. Toxins are labelled with their Swiss-Prot sequence identifiers except for bucandin from Malayan krait^26^ and TxS6C6 from eastern Jameson’s mamba^51^.

HPLC-purified toxin was reduced and alkylated with DTT (dithiothreitol) and iodoacetamide, respectively, and N-terminal sequencing of the intact peptide revealed the sequence of the first 46 amino acids up to Lys 46, with a single E/C ambiguity at position 39. MS/MS analysis on a 2001 Da LysC cleavage product confirmed the end of the Edman-derived sequence, clearing up the ambiguity at position 39 (see Supplementary Fig. S1). Additionally, LysC cleavage also yielded a product with a mass corresponding exactly to the remaining C-terminal region beyond K46. This product was purified by HPLC and used for further N-terminal sequencing, yielding the sequence from position 47 to 61. Ion-trap MS fragmentation of this peptide allowed for the identification of the remaining two residues (see Supplementary Fig. S2). The final peptide sequence for Tx7335 (see Fig. 2) consists of 63 amino acid residues and contains 8 cysteines, which, based on the mass of the native peptide, are all involved in disulphide bridges in the native structure. Sequence alignment revealed that Tx7335 belongs to the family of three-finger toxins (Fig. 2).

### Disulphide bonding pattern of Tx7335

**Figure 3:**
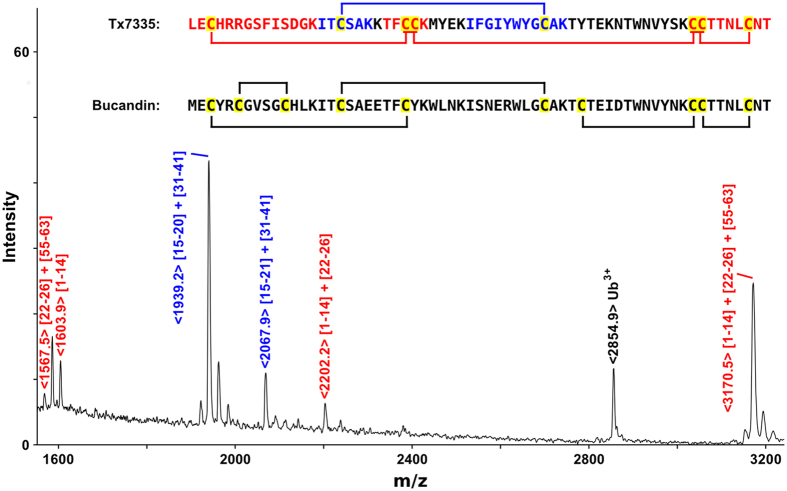
Determination of disulphide bond connectivities in Tx7335. A MALDI-TOF mass spectrum of LysC-cleaved native Tx7335 is shown, and peaks originating from cysteine-containing cleavage products are labelled. The peptide sequences of Tx7335 and bucandin are shown, and cysteine residues are highlighted in yellow. The Tx7335 sequence shows the Cys-containing peptide species in the same colours as the labels of the corresponding peaks in the mass spectrum. The bucandin sequence shows the disulphide connectivities as observed in the crystal structure^26^, and the Tx7335 sequence shows the disulphide bridges as experimentally determined with remaining ambiguities indicated.

The disulphide bridge connectivities were partially determined by complete LysC cleavage of native toxin. The resulting disulphide-connected peptides were identified by MALDI mass spectrometry (Fig. 3). Two major cysteine-containing cleavage products were observed. One peak of mass 1939.2 Da represents the disulphide-linked peptides I15-K20 and I31-K41. Since both of these peptides only contain one cysteine residue, it can be unambiguously determined that C17 is connected to C39. The other observed peak of mass 3170.5 Da is consistent with a product containing the linked L1-K14, T15-K20 and C55-T63 peptides. Since these three cleavage peptides contain all six remaining cysteines, further disulphide bridging information was obtained from the observation of a prompt fragmentation at the S-S bonds in the spectrum as well as from a separate ion-trap MS/MS analysis of the same 3170.5 Da species. In both cases, the L1-K14/T22-K26 and T22-K26/C55-T63 pairs were found, whereas no fragments containing linked L1-K14 and C55-T63 could be observed. This shows that C3 is connected to either C24 or C25, with the other cysteine in fragment T22-K26 forming a disulphide bond with C55, C56 or C61, and an additional disulphide bond existing between the other two cysteine residues in the C-terminal cleavage peptide (Fig. 3).

### Sequence homology of Tx7335 with other snake toxins

The sequence of Tx7335 consists of 63 residues, and sequence similarity searches indicate that it belongs to the three-finger snake toxin family (Fig. 2). The most closely related three-finger toxin is bucandin (61% amino acid identity) from the malayan krait (*Bungarus candidus*), a toxin that enhances presynaptic acetylcholine release through an unknown mechanism^26^.

### Functional characterisation of Tx7335

**Figure 4:**
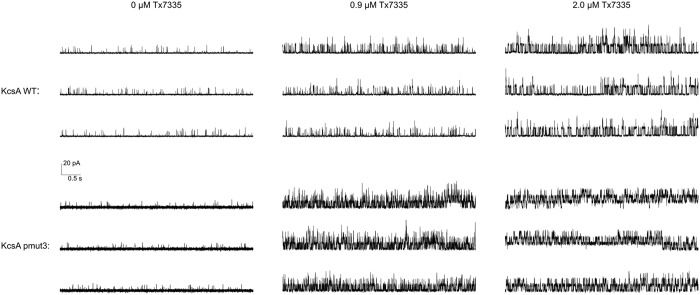
Single-channel traces of wild-type and mutant KcsA in artificial bilayers show the activating effect of adding the indicated concentrations of Tx7335 to the outside bath solution. Shown are 15 s representative traces of 2 minute recordings for each condition. All traces for each channel are from a single bilayer, and recordings were started 2 minutes after each addition of toxin.

**Figure 5:**
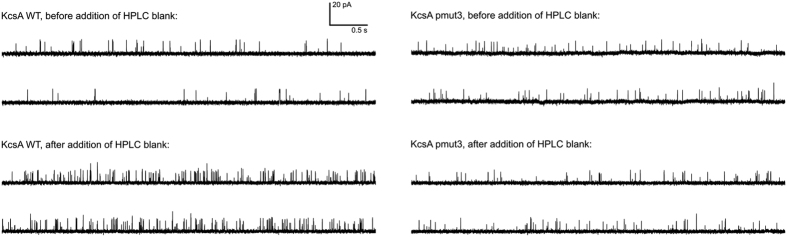
Single-channel KcsA traces upon addition of negative control samples (lyophilized HPLC baseline aliquots). Representative 10 s traces are shown for both WT and pmut3 KcsA bilayers before and after the addition of lyophilized HPLC baseline samples. The same bilayer is shown before and after addition of blank for each channel type.

In order to observe the functional effects of Tx7335 on channel activity, we reconstituted KcsA into planar lipid bilayers and measured the changes in single-channel activity upon addition of the toxin. Full-length KcsA was reconstituted into 3:1 1-palmitoyl-2- oleoylphosphatidylglycerol/1-palmitoyl-2-oleoylphosphatidylethanolamine (POPG/POPE) vesicles by dialysis. Lipid planar bilayers were painted with the same lipid composition, and the buffers were adjusted to pH 7 on the *cis* side and pH 4 on the *trans* side. Since KcsA only opens when the cytosolic C-terminal domain experiences low pH, this arrangement allows the observation of channels of a single orientation in an artificial bilayer^1^. The reconstitution concentration in vesicles was chosen such that only one to a few channels were present in each membrane. When we added Tx7335 to the outside bath, an increase in channel openings could be observed in a dose-dependent manner both for WT and pmut3 (see representative traces in Fig. 4, and all-point histograms in Supplementary Fig. S3). Mean open- and closed-time analyses of all traces were performed using the maximum interval likelihood (MIL) method with the software package QuB^27^, and results are summarised in Table 1 (data fits are shown in Supplementary Figs 4 and 5). It can be noted that the addition of 2.0 μM Tx7335 induces an ~ 8-fold increase in mean open times for wild-type KcsA and a ~ 13-fold increase for pmut3 KcsA (see Table 1). The correlated increases in open probability of about 40-fold for wild-type and about 90-fold for pmut3 channels reveal that there is an increase in the frequency of openings in addition to the increase in open times. Because of the presence of at least three (WT) or four (pmut3) individual channels in the analysed bilayers that only became evident after addition of toxin (see Fig. 4), the open probabilities cannot be directly interpreted as single-channel values. However, the relative differences of open probability in the absence and presence of toxin are a valid indicator of toxin effect since all toxin addition series are performed on the same bilayer. For calculation of the open time values, any segments of the traces that showed simultaneous opening of multiple channels were omitted from the MIL analysis. In order to ensure that the observed effect is due to the polypeptide itself and not an artefact due to some contaminating molecule from the HPLC purification, we collected an HPLC fraction of the same volume immediately following the Tx7335 peak. This fraction was lyophilized and re-solubilized in the same volume and buffer as the 200 μM toxin stock solution (leading to a solution of the same neutral pH as toxin stocks, since most TFA from the HPLC running buffer was removed during lyophylisation and any remaining traces were neutralized by the excess buffer in the stock solution). We performed this experiment both with WT and pmut3 KcsA, and showed that much smaller if any changes were observed in either case (Fig. 5, Supplementary Figs S6 and S7, and Table 1). While a roughly three-fold change in open probability was found with WT KcsA, this is much less than that observed in any experiments with toxin, and mean open times remained constant. With pmut3 KcsA, an even smaller increase in open probability of about 1.5-fold was observed with a mean open time increase of about 1.2-fold (see Table 1). Since the observed changes are at least an order of magnitude smaller than those seen with toxin and there is no significant effect on mean open times in these negative controls, we can conclude that the observed activating effect of the toxin is indeed due to the polypeptide and not an artefact of toxin purification or sample preparation. The small change in open probability that is observed for WT with HPLC blank is most likely due to normal variability in single-channel activity on the time scale of the experiment. We also attempted to add the toxin to the inside chamber to make sure that the observed activating effect is due to a specific interaction between toxin and extracellular channel surface. Indeed, toxin delivered to the inside did not cause any change in the channel open probabilities or mean open times (data not shown).

**Figure 6:**
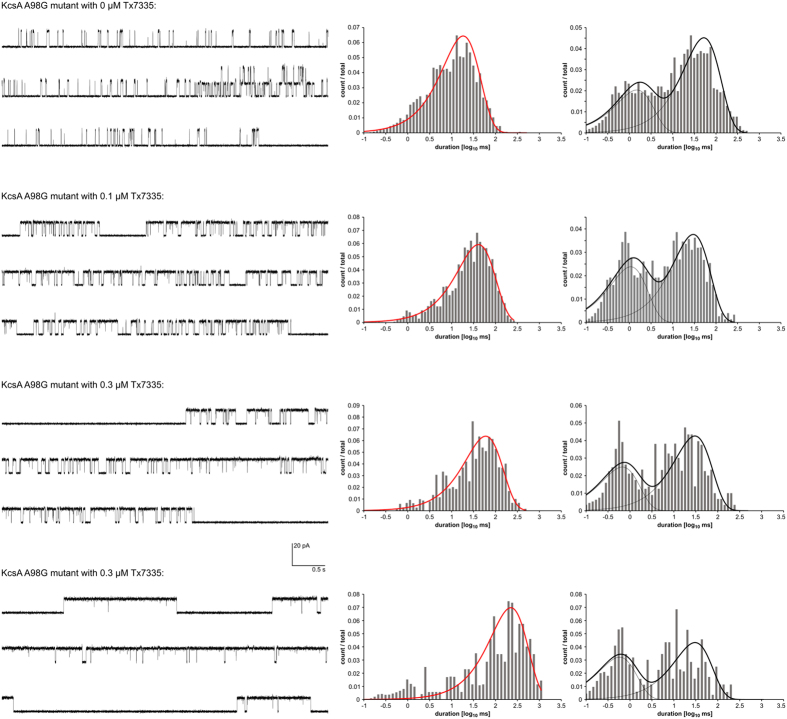
Single-channel traces and open-/closed-time histograms of A98G KcsA in the absence and presence of varying amounts of Tx7335. Representative 15 s traces as well as open- and closed-time histograms with their associated MIL fits are shown. For MIL fitting, the long, silent interburst segments as well as areas with 2 simultaneously open channels were removed from the analysis.

In addition to WT and pmut3 KcsA, we also tested the effect of Tx7335 on the KcsA A98G mutant, which shows significantly increased mean open times^28^ while retaining the ability to inactivate^29^. In this mutant, the addition of Tx7335 again induced a dose-dependent increase in mean open times, but because the single-channel traces showed long periods of inactivity between bursts of channel opening events, this did not correlate with a large effect on open probabilities (see Table 1, Fig. 6 and Supplementary Fig. S8). MIL analysis of the traces was performed omitting the long interburst periods. The traces could be fit with a model consisting of a single open and two closed states (Fig. 6). Within the tested toxin concentrations, the dependence of mean open times on the toxin concentration followed a roughly linear trend (Supplementary Fig. S9), indicating that saturation of the activating effect is not achieved with 0.9 μM toxin.

## Discussion

**Figure 7:**
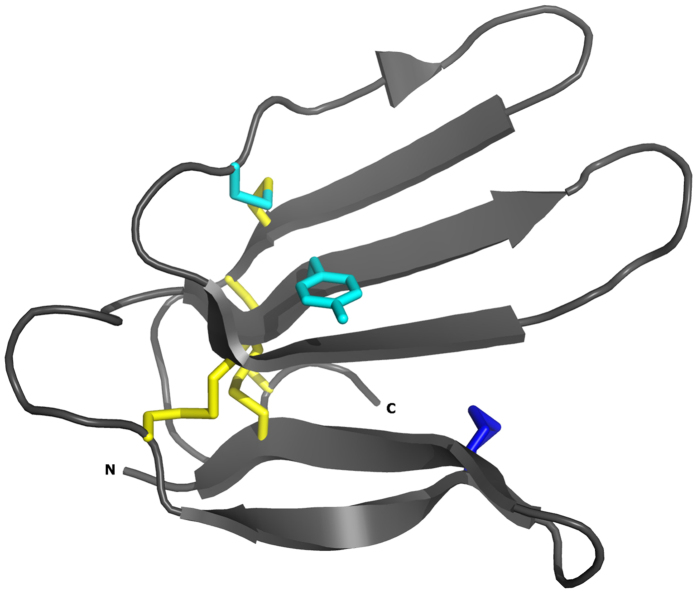
Ribbon diagram of the X-ray crystal structure of bucandin^26^. Cysteines that are conserved between bucandin and Tx7335 are shown in yellow. The extra N-terminal disulphide bridge that characterizes the long-form three-finger toxins is shown in blue. Y25 (which is a cysteine in Tx7335) and C43 (tyrosine in Tx7335) are shown in cyan. N- and C-termini are labelled.

We have identified and characterized a new mamba toxin Tx7335 from *Dendroaspis angusticeps* that acts on the bacterial potassium channel KcsA and has a unique channel-activating effect. Based on sequence homology, Tx7335 belongs to the family of the three-finger snake toxins, named after their characteristic fold that includes three beta hairpins sticking out like three fingers (Fig. 7). This family of snake toxins includes the α-neurotoxins (acetylcholine receptor inhibitors), the cardiotoxins (which damage cell membranes), the fasciculins (acetylcholine esterase inhibitors), the dendroaspins (antagonists of various cell adhesion processes) and L-type calcium channel blockers^30^. The potassium channel-activating function of Tx7335 we have observed is therefore very unusual for this family of toxins. The three-finger toxin family can also be sub-divided based on the sequence length and number of cysteine residues into the short-form toxins with 4 disulphide bonds and the long-form toxins that show an additional disulphide bond in the loop between first and second beta-strand^30^. Interestingly, although Tx7335 has the highest sequence similarity with members of the long-form three-finger toxins, it markedly differs from them in the number and positions of cysteines. First, Tx7335 has only 8 cysteines like the short-form toxins. Furthermore, it shows a unique variation in the cysteine scaffold, with the presence of tyrosine 43 at the site of a highly conserved cysteine in all three-finger toxins, whereas a cysteine is instead found at position 25 in Tx7335, which is a tyrosine in most other three-finger toxins (Fig. 2). The partial disulphide bridge mapping of Tx7335 is consistent with that found in bucandin and other three-finger toxins. Comparison with the structure of bucandin^26^,^31^ shows that cysteine 55 should be able to form a disulphide bridge with cysteine 25 without major disruptions in the structure due to the spatial proximity of the two residues, although this may lead to an increase in the flexibility of the “finger” between residues 43 and 55 that is stabilized by a disulphide bond in other three-finger toxins (Fig. 7). It is possible that this novel arrangement of linked cysteines in the sequence plays an important role in the unique functional effects observed for Tx7335.

To understand the functional mechanism of activation of KcsA by Tx7335, it is useful to briefly review the current state of knowledge on KcsA conformational gating: KcsA is activated by low intracellular pH conditions (pH range = 3.00–4.75) acting on the intracellular side of the channel^32^,^33^. pH-jump experiments in KcsA performed both at the ensemble and single-channel level showed a macroscopic activation of KcsA that is suggestive of multiple conformational states. These experiments also demonstrated that KcsA channels activate in the milliseconds range, and inactivate in the seconds timescale in the continuous presence of protons^16^. The various conformational transitions during activation are not voltage-dependent, whereas inactivation of KcsA is modulated by voltage and can occur from the fully open conducting channel or its partial states via a pH-independent manner^16^. Fluorescence lifetime measurements of the TM2 bundle openings have shown that the low open probability observed for KcsA under steady-state conditions in artificial bilayers is not explained by the TM2 movements but linked to this inactivation process^12^. Mutagenesis studies have pinpointed residues around the selectivity filter that are involved in this inactivation gate^34^,^35^, and recent crystal structures of a constitutively open channel^17^,^36^ and a non-inactivating mutant^37^, together with molecular dynamics simulations of structural transitions in the selectivity filter^38^,^39^, have shed light on the structural determinants of inactivation, in particular the importance of an extensive hydrogen-bonding network behind the selectivity filter. Given this mechanistic understanding, it makes sense to stipulate that Tx7335 functions by affecting KcsA inactivation, since the toxin acts from the extracellular side of the membrane, far from the intracellular pH gate.

To our knowledge, Tx7335 is the first peptide toxin that induces an increase in potassium currents via binding to the extracellular side of the potassium channel pore domain. It has however been reported that in the bacterial voltage-gated potassium channel KvLm, where the pore-blocker toxin charybdotoxin leads to a great reduction of single-channel currents, this is accompanied by a significant increase in open probability, which the authors attribute to a stabilization of the conductive conformation of the selectivity filter^40^. This link between pore blocker toxin binding and stabilization of the conductive selectivity filter conformation has also been observed in solid-state NMR and computational studies of the interaction between kaliotoxin and a toxin-sensitive KcsA mutant^41^,^42^, and may be a general feature of these toxins that is generally not observable due to the complete block of potassium currents in the toxin-bound state. A recent study linking the conformational and dynamic state of the outer vestibule to the inactivation state of the selectivity filter^43^ further suggests a mechanism by which toxin binding from the extracellular side can shift the equilibrium between conductive and inactivated states. Therefore, Tx7335 appears to increase KcsA channel openings by shifting the equilibrium between C-type inactivated and conductive conformations of the selectivity filter, either through stabilizing the conductive state or destabilizing the C-type inactivated conformation. We originally pulled down Tx7335 at neutral pH in detergent micelles, where the channel’s pH gate is closed but the selectivity filter is in the conductive state^2^. This demonstrates that Tx7335 strongly binds to the channel in the conductive state, suggesting that the stabilization of this state is the more likely mechanism of action of Tx7335.

The location of the A98G mutation is around the hinge region in TM2 that is involved in the C-terminal helix bundle opening in KcsA and other K^+^ channels^28^,^44^. The increased mean open times observed in this mutant are further evidence that the C-terminal helix bundle and hinge regions are indeed allosterically linked to the selectivity filter inactivation gate. The fact that even this mutant is further activated by Tx7335 indicates that there is a mechanistic difference between the allosteric effects induced by toxin binding from the extracellular side and those due to interaction with the intracellular hinge and pH gate. Mechanistically, we postulate that binding of the toxin to the extracellular turret region induces conformational or dynamic changes that are allosterically coupled to the selectivity filter. The fact that both the WT and pmut3 forms of the channel are susceptible to the toxin, with only a slight increase in toxin susceptibility for the charybdotoxin-sensitive mutant, suggests that there is only a small overlap in binding sites between the pore-blocker scorpion toxins and Tx7335. However, further structural and mutational studies will be required to elucidate the details of the Tx7335 binding site and detailed mechanism of activation.

The newly identified potassium channel-activating mamba toxin Tx7335 will be a useful tool to obtain more information on the mechanism of slow inactivation in KcsA and potentially other potassium channels. In particular, it will allow the experimental determination of how conformational or dynamic changes at the extracellular surface of KcsA are linked to the selectivity filter conductive state. Previous NMR studies have implied the existence of an allosteric link between the pH gate and the inactivation region^45^,^46^, but Tx7335 will offer a new tool for probing a connection of this allosteric pathway to the extracellular surface. Furthermore, Tx7335 may also serve as the starting compound for the development of a new class of drugs against diseases caused by lack-of-function mutations, for which channel-activating molecules represent a potential break-through approach.

## Methods

### Expression and purification of KcsA

Expression plasmids for wild-type and pmut3 (Q58A, T61S, and R64D) KcsA with a 6xHis tag cloned into the pQE60 vector were obtained from Prof. Roderick MacKinnon and used to express and purify the channels as previously described^2^,^25^. In brief, channels were expressed in Xl1-blue *E. coli* cells, extracted from the membrane with 40 mM DM, purified on a TALON Co^2+^ resin and exchanged into a final buffer of 5 mM DM, 50 mM TRIS pH 7.5, 150 mM KCl for reconstitution into lipid vesicles or toxin pull-down assays.

### Toxin pull-down assay

10 mg of crude lyophilized *Dendroaspis angusticeps* venom (Latoxan) was dissolved in 1 ml of equilibration buffer (5 mM DM, 50 mM TRIS pH 7.5, 150 mM KCl), filtered, and applied to a 500 μl TALON Co^2+^ column. The venom was then eluted with an additional 4 ml of the same buffer, and the combined flow-through was collected and used as the pre-depleted venom fraction in the following pull-down assay: Two 200 μl TALON Co^2+^ columns were pre-equilibrated with equilibration buffer, and one of the columns was loaded with 1.7 mg of channel in equilibration buffer at a protein concentration of 0.5 mg/ml, whereas the other column was left empty and used as a negative control. Both columns were washed with 2 ml of equilibration buffer, and then loaded with half of the pre-depleted venom fraction each. Both columns were then washed with 5 ml of equilibration buffer, followed by 2.5 ml of 5 mM DM, 50 mM TRIS pH 7.5, 500 mM KCl and 40 mM imidazole. Any bound channel and peptides were then eluted from each column with 0.8 ml of 5 mM DM, 50 mM TRIS pH 7.5, 150 mM KCl and 400 mM imidazole.

### Mass spec analysis of pull-down assay eluates

Eluates were then analysed by MALDI-TOF using the ultrathin-layer sample preparation technique^47^. Eluates were diluted without further purification in a 1:20 ratio into a saturated solution of α-cyano-4-hydroxycinnamic acid in 3:1:2 formic acid:water:isopropanol and spotted onto a MALDI target pre-coated with an ultrathin layer of the same matrix. As soon as the sample showed a homogenous crystal layer, the leftover drop was removed, and the crystallised layer was washed with 5 μl of 0.1% trifluoroacetic acid (TFA) for a few seconds. MALDI-TOF spectra were recorded on a Voyager-DE STR spectrometer (PE Biosystem, Foster City, CA) operating in linear, delayed extraction mode.

### HPLC purification of Tx7335 from pull-down eluate and crude venom

HPLC analysis was performed on an Agilent 1100 series instrument using either a Vydac C18 column (5 μm, 4.6 × 250 mm for analytical runs) at a flow rate of 1 mL/min or an Agilent Zorbax C18 column (5 μm, 9.4 × 250 mm for semi-preparative runs) at a flow rate of 3 ml/min, using a two-component mobile phase system in which mobile phase A is 0.1% TFA in water and mobile phase B is 90% acetonitrile and 0.1% TFA in water. Absorbance was monitored at 214 and 280 nm. 400 μl of pull-down eluate were acidified with 2% TFA and injected on the above column. Peptides were separated with a 0–73% B gradient run over 40 minutes, and collected fractions were analysed by MALDI-TOF as above. For purification of Tx7335 from crude venom, 20 mg of crude venom were dissolved in 0.5 ml of 50 mM TRIS pH 7.5, 150 mM KCl, filtered, then purified on a semi-prep HPLC column using the following gradient: 0–35% B in 20 min, followed by 35–55% B in 40 min and 55–90% B in 10 min.

### Sequence determination of Tx7335

Reduction and alkylation was carried out on native Tx7335 in 40% acetonitrile, 50 mM TRIS pH 7.5 by incubation with 5 mM DTT at 42 °C for 5 min, followed by addition of 20 mM iodoacetamide and incubation at 25 °C for 20 min. Reduced and alkylated Tx7335 was then re-purified by HPLC for sequencing by Edman degradation (Rockefeller University Protein/DNA Technology Center). Protease cleavage experiments were performed on reduced and alkylated Tx7335 in crude reaction mixture by addition of 10 μg/ml endoproteinase Lys-C (Promega, sequencing grade). Proteolysis products were analysed either directly by MALDI-ion trap MS/MS (in 2,5-dihydroxybenzoic acid matrix in 60% methanol, 2% acetic acid in water on a Thermo Finnigan LCQ-DECAXP mass spectrometer with a homemade MALDI source^48^) or purified by HPLC after stopping the cleavage with TLCK and pepstatin A. Purified cleavage products were sequenced by Edman degradation or ESI-Ion trap MS/MS (on a Thermo LCQ-DECAXP + instrument). For the determination of disulphide connectivities, native Tx7335 in 30% acetonitrile, 50 mM TRIS pH 7.5 was cleaved with Lys-C in a 5:1 molar toxin:protease ratio at 25 °C for 16 h, and fragments were analysed either by MALDI-TOF or MALDI-ion trap as above.

### Electrophysiology

KcsA reconstitution and single-channel analysis was performed as previously described by Valiyaveetil *el al.*^49^. Purified KcsA was reconstituted into 3:1 POPE:POPG vesicles at a 1:400 protein:lipid mass ratio in a reconstitution buffer of 10 mM HEPES pH 7.0, 450 mM KCl, and 4 mM N-methyl-D-glucamine. Bilayers of the same lipid composition were formed over a 300 μm diameter hole in a polystyrene partition between two buffer chambers, and fusion of vesicles was induced by a potassium gradient across the bilayer. Upon fusion, the KCl concentrations were adjusted to 150 mM on both sides of the membrane. The pH values of 7.0 (*cis*) and 4.0 (*trans*) were maintained by 10 mM of HEPES and succinate buffers, respectively. The membrane voltage was controlled to +100 mV and the current recorded by an Axopatch 200B amplifier with a Digidata 1322A analog-to-digital converter and Axoclamp software (Molecular Devices)^1^,^50^. BSA at a concentration of 0.5 mg/ml was added to all solutions prior to addition of Tx7335 to prevent non-specific binding of the toxin to any surfaces. Toxin stock solutions at 200 μM concentration were prepared from lyophilized HPLC Tx7335 fraction, either in the *cis* or *trans* buffers for addition to the respective chambers.

## Additional Information

**Accession codes:** The protein sequence data for Tx7335 reported in this paper will appear in the UniProt Knowledgebase under the accession number C0HJT4.

**How to cite this article**: Rivera-Torres, I. O. *et al*. Discovery and characterisation of a novel toxin from *Dendroaspis angusticeps*, named Tx7335, that activates the potassium channel KcsA. *Sci. Rep.* **6**, 23904; doi: 10.1038/srep23904 (2016).

## Supplementary Material

Supplementary Information

## Figures and Tables

**Table 1:** Single-channel analysis of WT and mutant forms of KcsA in the absence and presence of variable concentrations of toxin.

	Number of events	P_o_ (%)	Single-channel conductance (pS)	τ_c_ (ms)	τ_o_ (ms)
WT KcsA (no Tx)	1618	0.76	96 ± 23	145	1.06
WT KcsA (0.9 μM Tx)	6795	11.6	126 ± 45	26.6	3.40
WT KcsA (2.0 μM Tx)	4883	42.3	128 ± 17	12.4	9.06
pmut3 KcsA (no Tx)	2436	0.74	87 ± 24	100	0.64
pmut3 KcsA (0.9 μM Tx)	6922	39.6	99 ± 22	6.02	3.91
pmut3 KcsA (2.0 μM Tx)	5079	58.1	91 ± 20	5.91	8.25
A98G KcsA	4511	36.4	115 ± 20	1.16/30.2	13.2
A98G KcsA (0.1 μM Tx)	1496	67.8	115 ± 17	0.99/27.5	37.8
A98G KcsA (0.3 μM Tx)	597	74.5	119 ± 17	0.62/27.7	54.9
A98G KcsA (0.9 μM Tx)	349	92.1	120 ± 18	0.53/27.6	204
WT KcsA (no blank)	1577	0.86	95 ± 23	151	1.21
WT KcsA (plus blank)	4745	2.31	88 ± 28	49.2	1.19
pmut3 KcsA (no blank)	1633	0.69	84 ± 32	129	0.80
pmut3 KcsA (plus blank)	2173	1.09	84 ± 27	97.5	0.98

Statistics of single-channel analysis of all traces presented in the paper are shown here.

In all cases, traces were edited before analysis to remove regions with multiple simultaneously open channels. For A98G traces, interburst regions without any channel openings lasting longer than 1 s were also removed from analysis. The open probability P_o_ and the single-channel conductance were obtained from half-maximum amplitude idealization, and the mean open- and closed-times τ_o_ and τ_c_ from MIL fitting. Values have been derived from a single 2 min. trace for each condition. The given errors in the single-channel conductances are based on the combined standard deviations in actual amplitude values for the open and closed states. A simple two-state model was used in MIL fitting except for A98G, which was modelled with two closed and one open state.
