# Supplementary material for: Discovery and characterisation of a novel toxin from Dendroaspis angusticeps, named Tx7335, that activates the potassium channel KcsA
Source: Sci Rep. 2016 Apr 5;6:23904. doi: 10.1038/srep23904 (PMC4820689; doi:10.1038/srep23904)
Supplement: Supplementary Information [file srep23904-s1.pdf]

Supplementary information for:

# Discovery and characterization of a novel toxin from *Dendroaspis angusticeps*, named Tx7335, that activates the potassium channel KcsA

**Iván O. Rivera-Torres[2,3], Tony B. Jin[1], Martine Cadene[4,5], Brian T. Chait[4], and Sébastien F. Poget[1*]**

[1]From the Department of Chemistry, CUNY Graduate Center and Institute for Macromolecular Assemblies
College of Staten Island, City University of New York, Staten Island, NY 10314

[2]LaGuardia Community College, City University of New York, Long Island City, NY 11101

[3]Current address: Salish Kootenai College, Pablo, MT 59855

[4]The Rockefeller University, New York, NY 10065

[5]Current address: Center for Molecular Biophysics, CNRS UPR4301, 45071 Orléans, France

*To whom correspondence should be addressed - Tel.: (718) 982 4183, Fax: (718) 982 3910, E-mail: sebastien.poget@csi.cuny.edu

**Content:**

**Supplementary Figure S1**
**Supplementary Figure S2**
**Supplementary Figure S3**
**Supplementary Figure S4**
**Supplementary Figure S5**
**Supplementary Figure S6**
**Supplementary Figure S7**
**Supplementary Figure S8**
**Supplementary Figure S9**

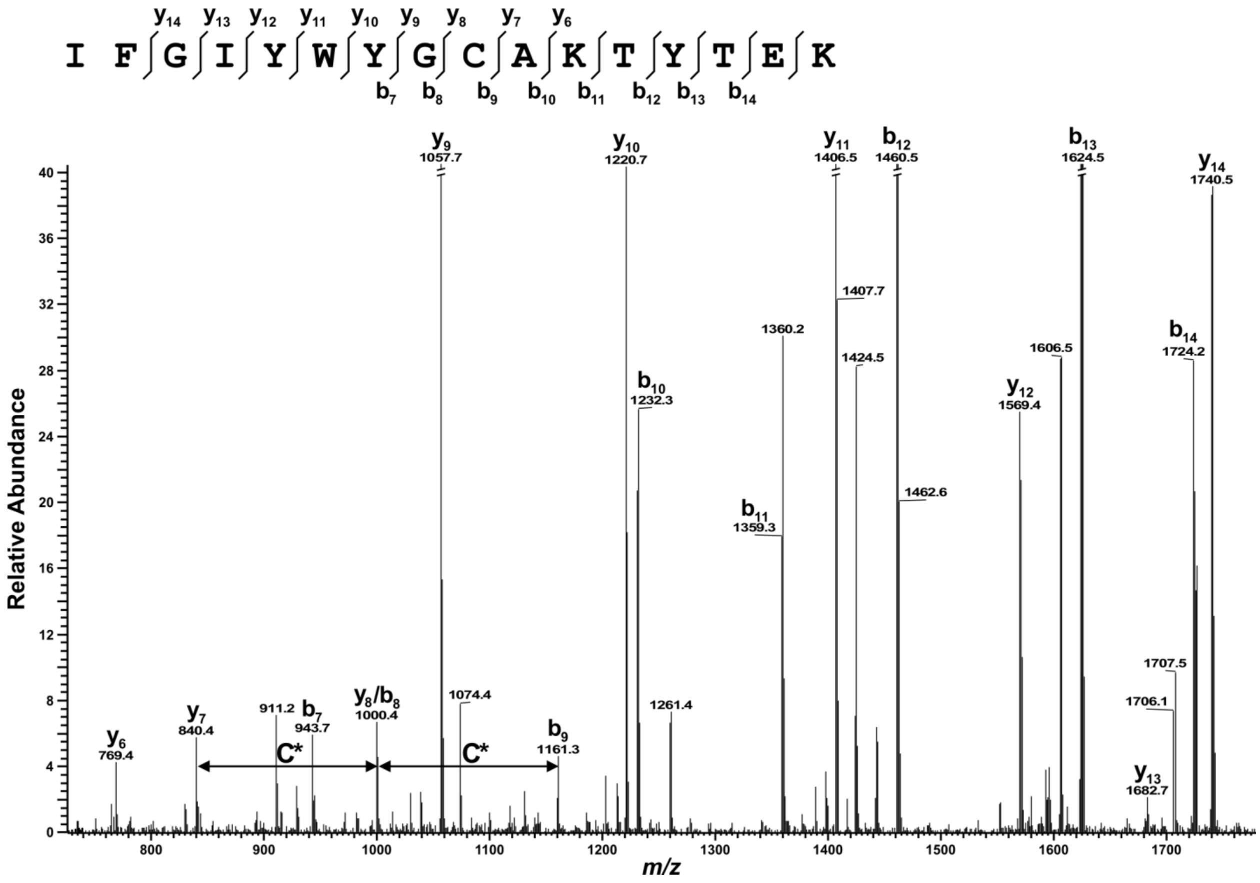


**Supplementary Figure S1.** MALDI-ion trap fractionation of the 2001 Da Tx7335 LysC cleavage product (I31-K46 of the Tx7335 sequence). Fragment ions of the y and b series are labeled in the spectrum and their position is indicated on the amino acid sequence of the peptide. The arrows in the spectrum indicate the spacing between $y_7$ and $y_8$ and between $b_8$ and $b_9$, both of which correspond to the mass of an S-carbamidomethyl cysteine and confirm the presence of a cysteine residue at position 39.

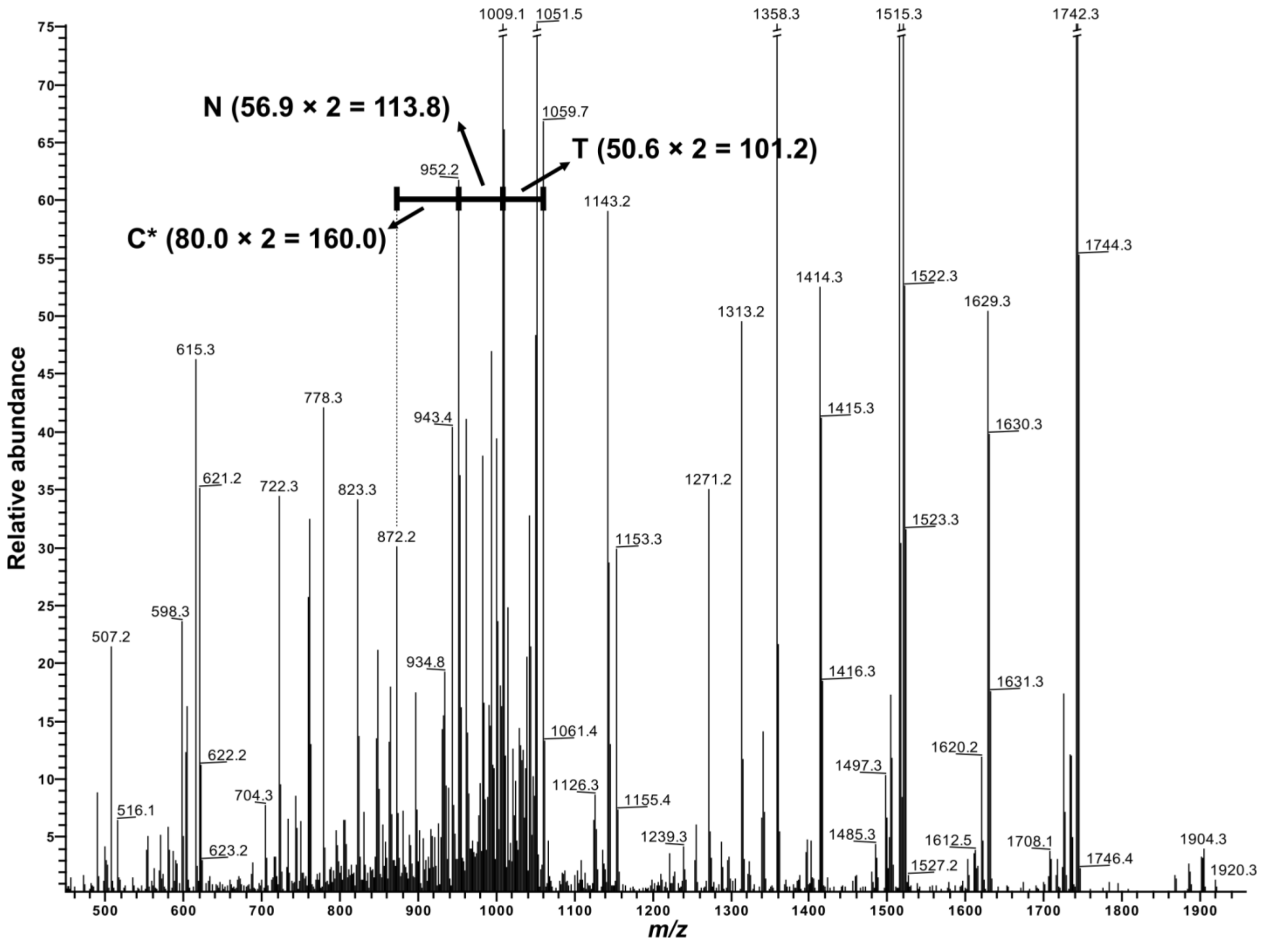


**Supplementary Figure S2.** ESI-ion trap MS/MS analysis of the 2137 Da C-terminal LysC cleavage product. The doubly-charged ion was selected as a precursor, and mass differences between the three largest b ions are highlighted, confirming the identity of the last three residues in the sequence as CNT.

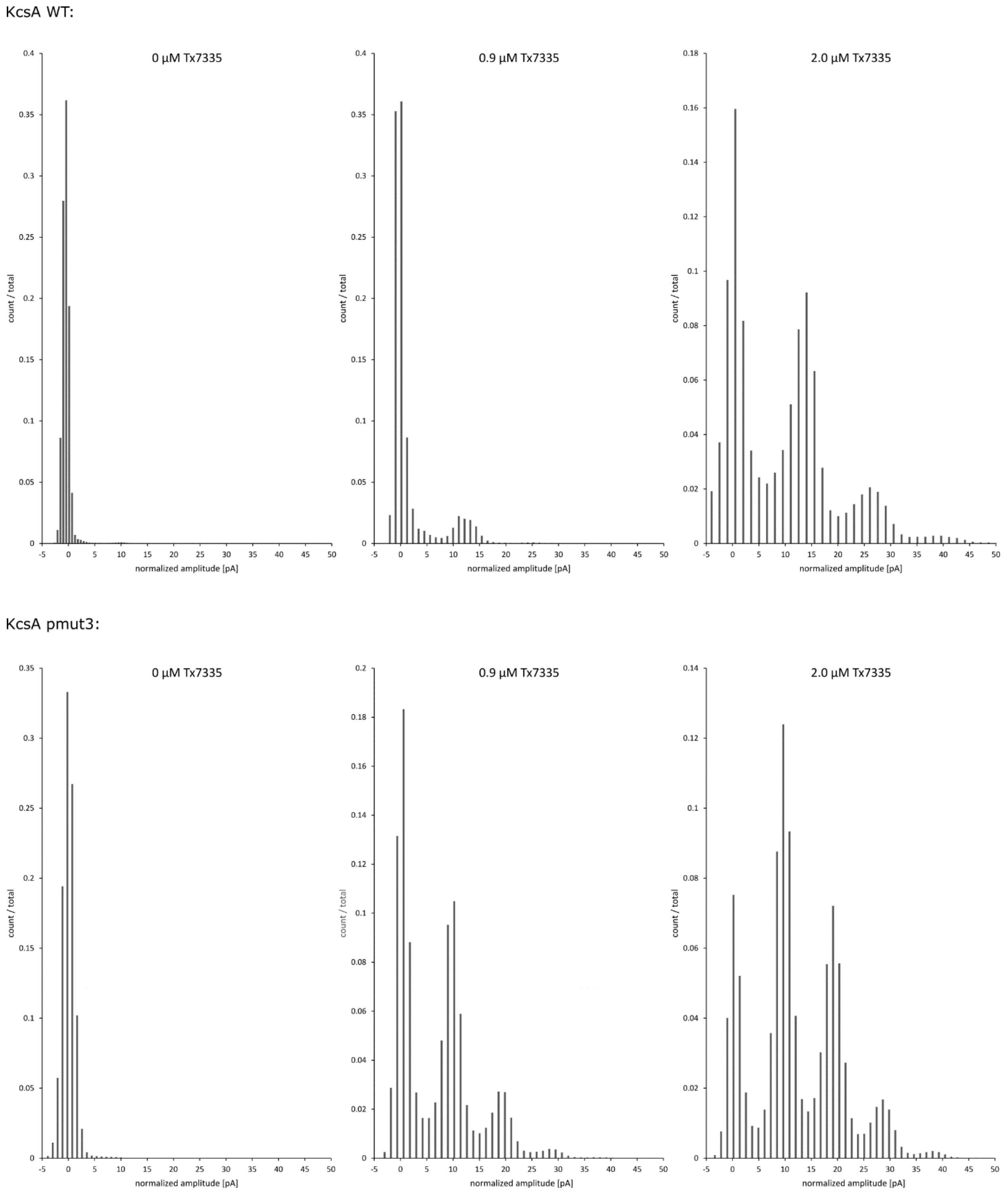


**Supplementary Figure S3.** All-point histograms of wild-type and pmut3 KcsA single-channel recordings in the absence and presence of Tx7335, showing the significant increase of higher amplitude states corresponding to 1-3 open channels (1-4 open channels for pmut3) upon addition of Tx7335. All histograms are derived from 2 min. recordings, and amplitudes were normalized to account for baseline drift over the course of multiple recordings on the same bilayer.

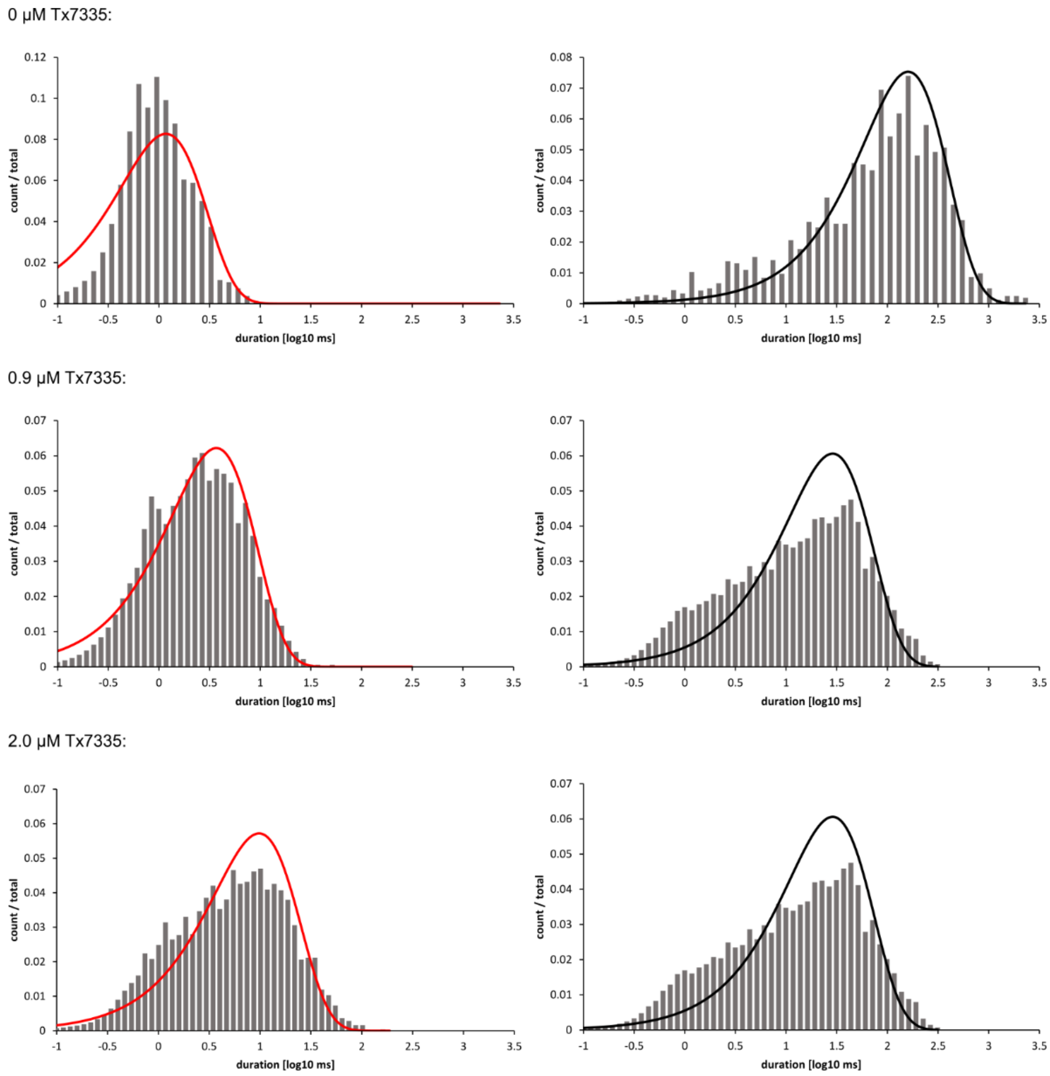


**Supplementary Figure S4.** Open- and closed-time histograms with their associated maximum interval likelihood fits are shown for wild-type KcsA single-channel recordings in the absence and presence of Tx7335. Histograms were derived from 2 min. recordings, with areas of the recordings with amplitudes corresponding to multiple concurrently open channels removed before the analysis.

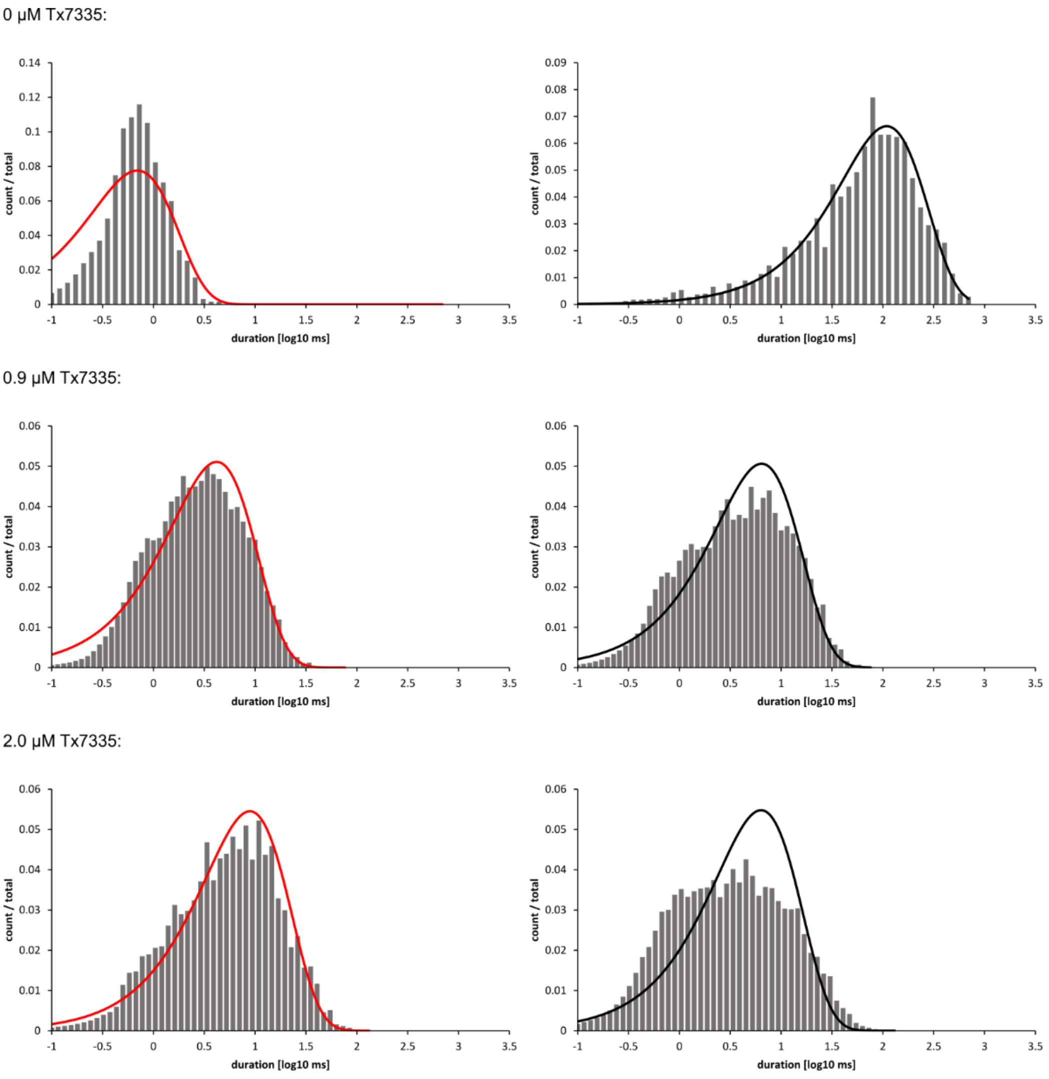


**Supplementary Figure S5.** Open- and closed-time histograms with their associated maximum interval likelihood fits are shown for pmut3 KcsA single-channel recordings in the absence and presence of Tx7335. Histograms were derived from 2 min. recordings, but areas of the recordings with amplitudes corresponding to multiple concurrently open channels were removed before the analysis.

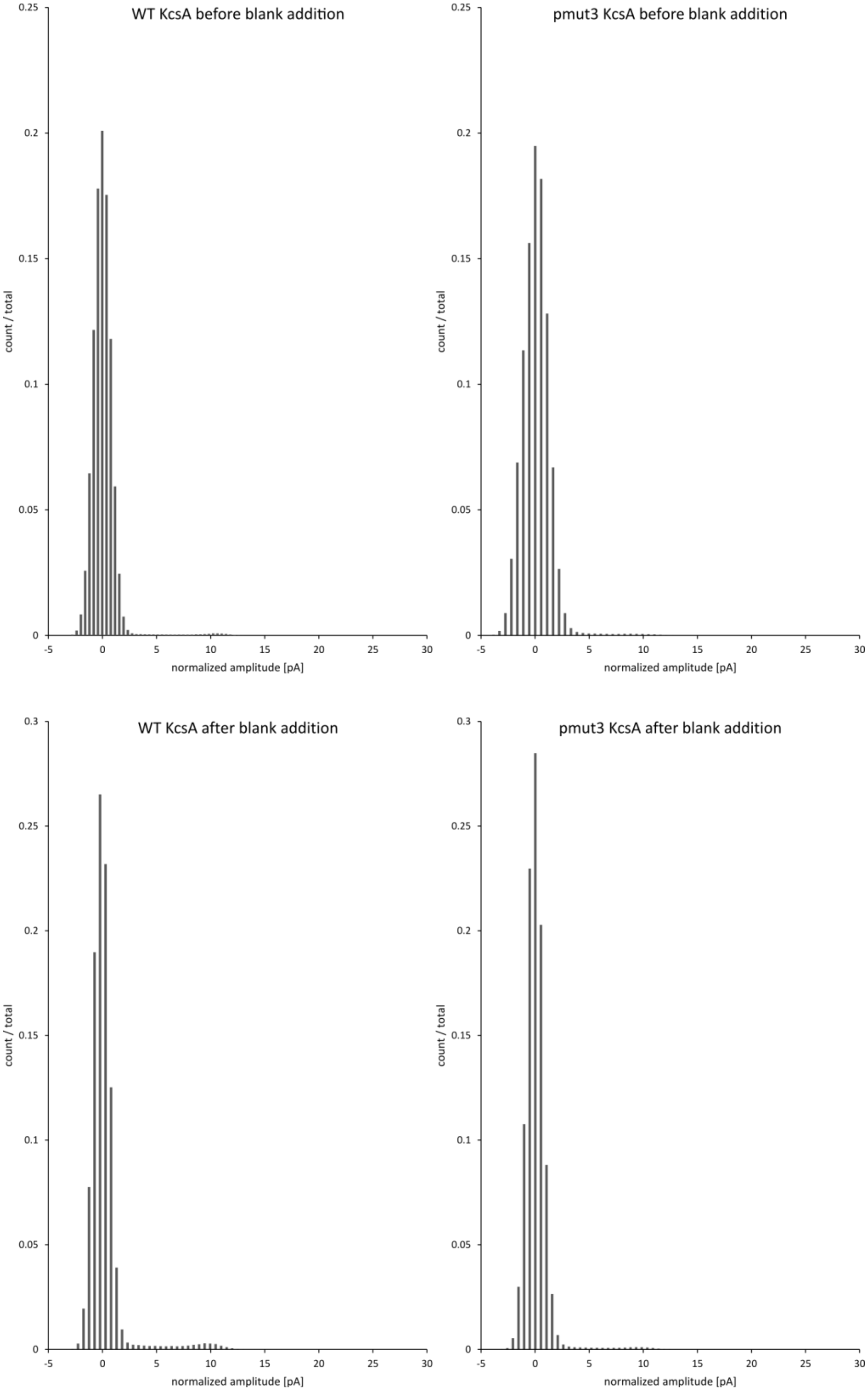


**Supplementary Figure S6.** All-point histograms of WT and pmut3 KcsA single-channel recordings before and after addition of HPLC blanks. All histograms are derived from 2 min. recordings.

WT KcsA before addition of HPLC blank:

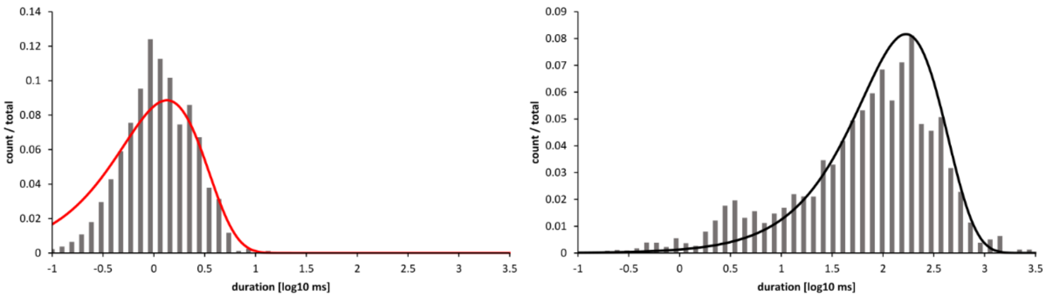


WT KcsA after addition of HPLC blank:

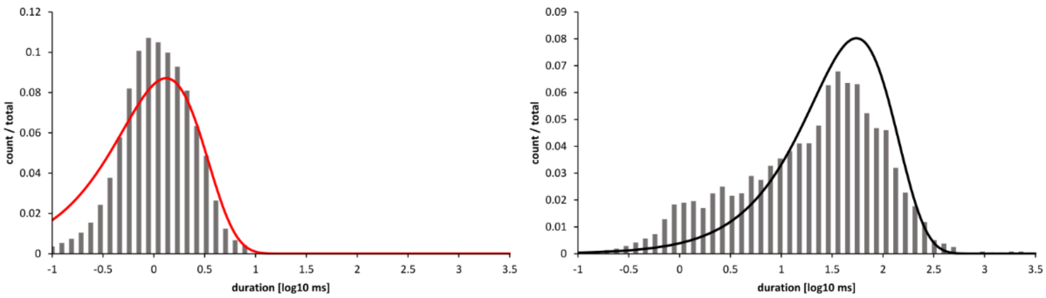


pmut3 KcsA before addition of HPLC blank:

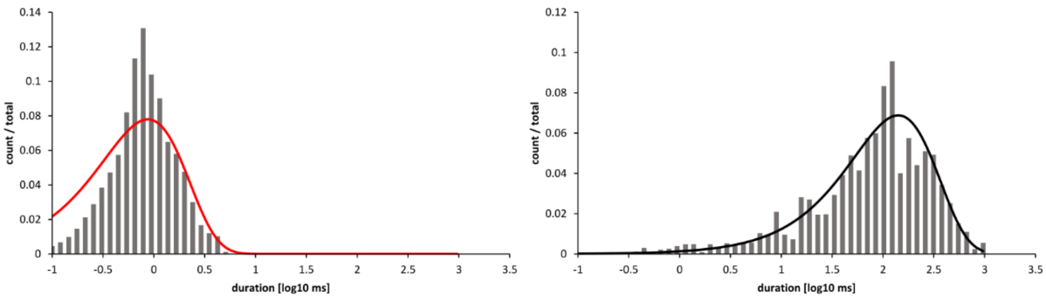


pmut3 KcsA after addition of HPLC blank:

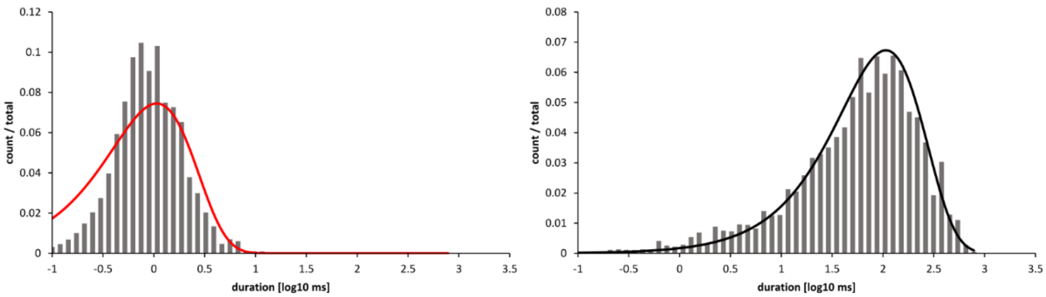


**Supplementary Figure S7.** Open- and closed-time histograms with their associated maximum interval likelihood fits are shown for WT and pmut3 KcsA single-channel recordings in the absence and presence of lyophilized HPLC blanks collected immediately following the Tx7335 peak. Histograms were derived from 2 min. recordings.

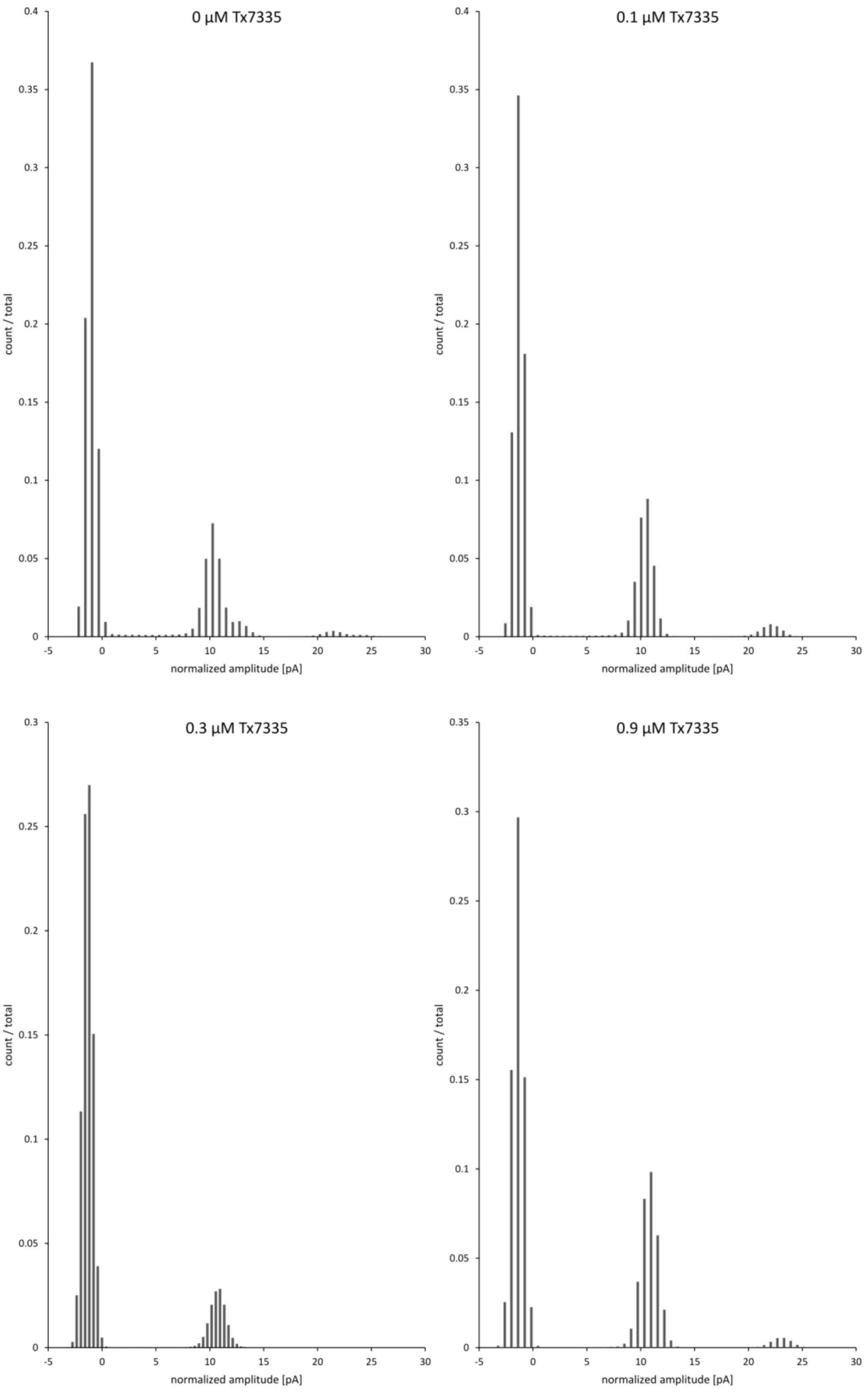


**Supplementary Figure S8.** All-point histograms of A98G KcsA single-channel recordings in the absence and presence of Tx7335. All histograms are derived from 2 min. recordings.

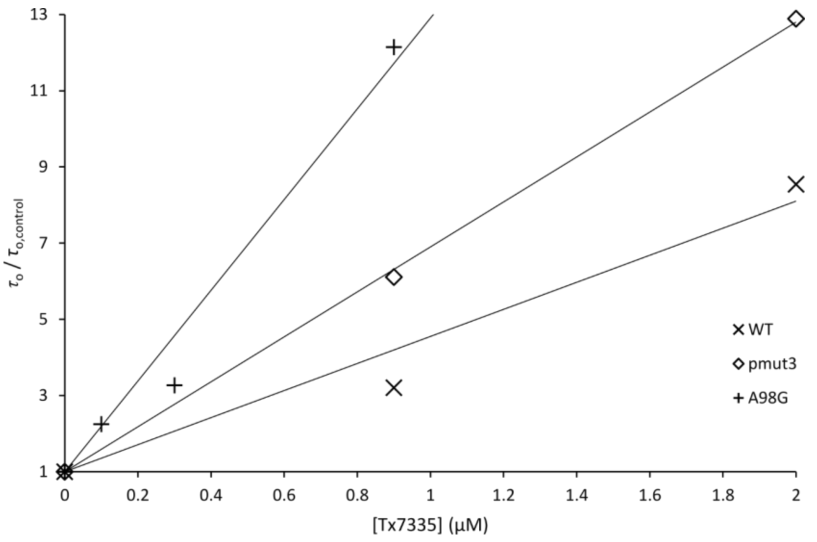


**Supplementary Figure S9. Dependence of mean open times on Tx7335 concentration for WT and mutant forms of KcsA.** The relative increase in mean open times upon addition of Tx7335 is plotted versus toxin concentration for WT, pmut3 and A98G KcsA. The linear fits are shown for reference only and have no physical meaning.
